# Norepinephrine depleting toxin DSP-4 and LPS alter gut microbiota and induce neurotoxicity in α-synuclein mutant mice

**DOI:** 10.1038/s41598-020-72202-4

**Published:** 2020-09-14

**Authors:** Sheng Song, Jie Liu, Feng Zhang, Jau-Shyong Hong

**Affiliations:** 1 Neuropharmacology Section, Neurobiology Laboratory, National Institute of Environmental Health Sciences, National Institutes of Health, Research Triangle Park, NC 27709 USA; 2 Key Lab for Basic Pharmacology of Ministry of Education and Joint International Research Laboratory of Ethnomedicine, Zunyi Medical University, Zunyi, 563000 Guizhou China

**Keywords:** Microbiology, Neuroscience, Systems biology, Neurology

## Abstract

This study examined the genetic mutation and toxicant exposure in producing gut microbiota alteration and neurotoxicity. Homozygous α-synuclein mutant (SNCA) mice that overexpress human A53T protein and littermate wild-type mice received a single injection of LPS (2 mg/kg) or a selective norepinephrine depleting toxin DSP-4 (50 mg/kg), then the motor activity, dopaminergic neuron loss, colon gene expression and gut microbiome were examined 13 months later. LPS and DSP-4 decreased rotarod and wirehang activity, reduced dopaminergic neurons in substantia nigra pars compacta (SNpc), and SNCA mice were more vulnerable. SNCA mice had 1,000-fold higher human SNCA mRNA expression in the gut, and twofold higher gut expression of NADPH oxidase (NOX2) and translocator protein (TSPO). LPS further increased expression of TSPO and IL-6 in SNCA mice. Both LPS and DSP-4 caused microbiome alterations, and SNCA mice were more susceptible. The altered colon microbiome approximated clinical findings in PD patients, characterized by increased abundance of *Verrucomicrobiaceae*, and decreased abundance of *Prevotellaceae*, as evidenced by qPCR with 16S rRNA primers. The Firmicutes/Bacteroidetes ratio was increased by LPS in SNCA mice. This study demonstrated a critical role of α-synuclein and toxins interactions in producing gut microbiota disruption, aberrant gut pro-inflammatory gene expression, and dopaminergic neuron loss.

## Introduction

Parkinson’s diseases (PD) is a common neurodegenerative disease characterized by loss of dopaminergic neurons in the substantia nigra pars compacta (SNpc), and by α-synuclein containing Lewy bodies^1^. PD development and progression are associated with chronic inflammation not only in the brain but also in the gut^2–5^. A stage-development of PD model showed that α-synuclein aggregates were initially found in the intestine, progressed to the medulla oblongata, and then to the SNpc, and finally to the neocortex^1,6,7^.

Enteric nervous system (ENS) is an integrative network of neurons in the intestinal wall^4,7^, and α-synuclein mutant (SNCA) mice that overexpress human A53T protein exhibited early and persistent expression of phosphorylated α-synuclein in the ENS^8,9^. Lipopolysaccharide (LPS)-induced PD model showed pathological α-synuclein aggregation in the large intestine with intestinal permeability changes in a manner similar to that observed in PD patients^10^.

Gut microbiota is now increasingly recognized to form the gut-brain-microbiota axis as a new paradigm in neurological and psychiatric disorders. Emerging evidence links perturbations in the gut microbiota to PD risk, onset, and progression^11–15^. For example, decreased *Prevotellaceae* was reported in PD patients^16–19^. PD patients with irritable bowel syndrome (IBS) had a lower fecal abundance of *Prevotella* bacteria than those without IBS-like symptoms^20^. Increased *Enterobacteriaceae* is associated with the severity of postural instability and gait difficulty^18^. *Verrucomicrobia* was also shown to increase in some PD patients^19,21^. Decreases in *Lachnospiraceae* and increased *Bifidobacteriaceae* were observed in Chinese PD patients^22^.

Our previous studies demonstrated that LPS-induced chronic inflammation causes progressive neurodegeneration in brain along with motor dysfunction that recapitulate neuropathological and symptomatic features of PD patients^2,23^. The selective norepinephrine (NE)-depleting toxin N-(2-chloroethyl)-N-ethyl-2-bromobenzylamine (DSP-4) also produced a time-dependent degenerative pattern similar to that generated by LPS^24^. Noradrenergic dysfunction produced by DSP-4 also enhances LPS-induced dopaminergic neuron loss in the SNpc^25^, and accelerates LPS-elicited inflammation-related neurodegeneration in an ascending sequential manner, together with behavioral dysfunctions^26^. However, little is known about the alterations of gut microbiota in LPS or DSP-4 induced chronic PD mouse models and whether gut microbiome alternations in our mouse PD models could mimic the findings in PD patients remains unclear.

Genetic mutation interacts with environmental factors to play a role in many diseases including PD. Since neuroinflammation and α-synuclein dysfunction potentiate each other to drive the “vicious cycle” of neuroinflammation^27^. The “two-hit” models, LPS or neurotoxin DSP-4 in combination with α-synuclein mutation, were used to examine alterations in gut microbiome, colon gene expression, behavioral impairments and dopaminergic neuron loss.

## Results

### General health and animal body weights

None of the SNCA and WT mice presented overt health impairment after the injection of the tolerant doses of LPS or DSP-4. Animal body weights were recorded weekly, and 13 months later, the body weights in WT mice were 43.4 ± 5.4, 38.3 ± 1.5, and 45.8 ± 3.7 g for Control, LPS, and DSP-4, respectively; the body weights in SNCA mice were 39.6 ± 3.7, 41.8 ± 1.6, and 44.9 ± 3.8 g for Control, LPS, and DSP-4, respectively. Meanwhile, behavioral tests and immunohistochemistry analysis were performed at this end time point to evaluate if mice displayed PD-like behavioral deficits and neuronal loss in SNpc.

### Impaired motor activity

**Figure 1:**
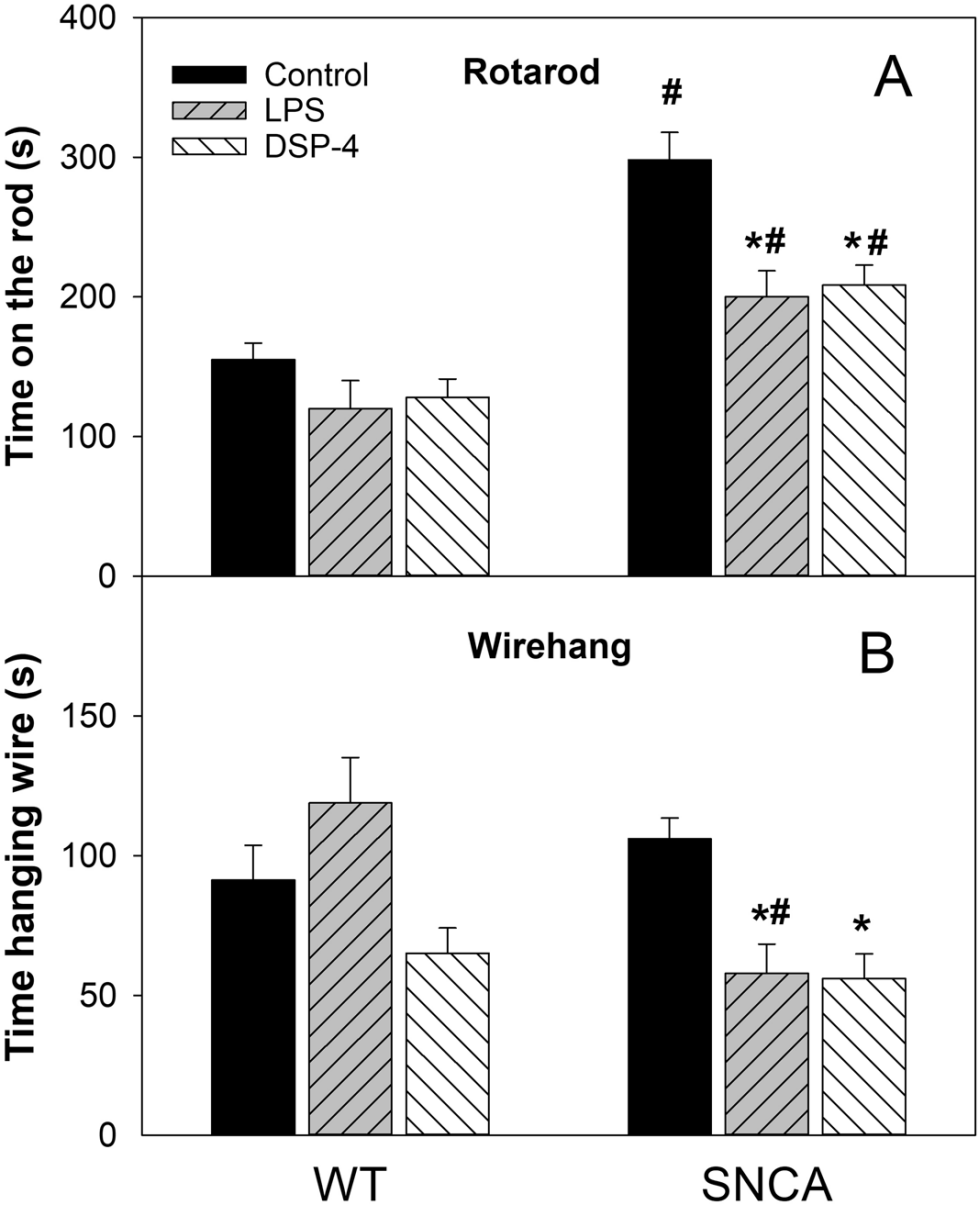
Behavioral tests. (**A**) Rotarod tests; (**B**) Wirehang tests. Mice were given a single injection of LPS (2 mg/kg) or DSP-4 (50 mg/kg), and behavioral tests were performed on 13 months after administration, respectively. Data are mean ± SE of 4–7 animals. *Significantly different from Controls, *p* < 0.05. ^#^Significantly different from WT mice, *p* < 0.05.

The behavioral activities were examined at 13 months after LPS and DSP-4 injection in WT and SNCA mice. Rotarod test is one of the classic methods to measure the muscular coordination in PD animals. As shown in Fig. 1A, LPS and DSP-4 significantly decreased Rotarod activity in SNCA mice, but only showed a trend of decrease in WT mice without statistically difference. The time stay on the rod for WT mice were 115 ± 12, 120 ± 20, and 128 ± 14 s for Control, LPS, and DSP-4, respectively; The time stay on the rod for SNCA mice were 298 ± 20, 200 ± 19, and 208 ± 15 s for Control, LPS, and DSP-4, respectively. Figure 1B shows that LPS and DSP-4 markedly decreased grip strength in SNCA mice, while showed no significant difference between groups in WT mice. The time hanging on the wire for WT mice were 91 ± 13, 119 ± 16, and 65 ± 9 s for Control, LPS, and DSP-4, respectively; The time hanging on the wire for SNCA mice were 106 ± 8, 58 ± 10, and 56 ± 9 s for Control, LPS, and DSP-4, respectively. Motor activities were decreased in LPS- and DSP-4 treated SNCA mice.

### Neurodegeneration

**Figure 2:**
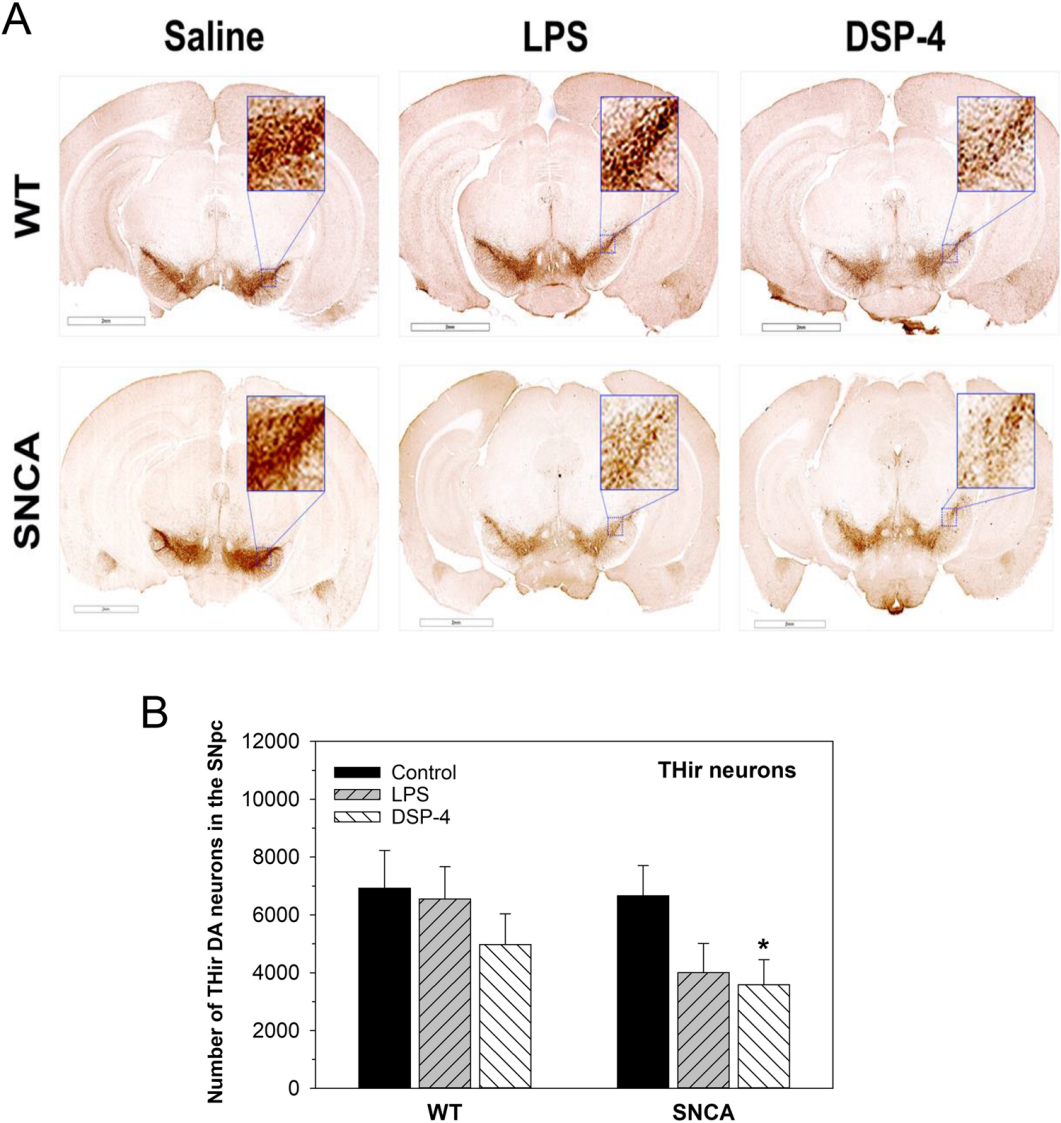
Immunohistochemistry. Mice were given a single injection of LPS (2 mg/kg) or DSP-4 (50 mg/kg), and brains were collected 13 months after administration. (**A**) Brain sections stained with anti-TH antibody (magnitude 4X), the THir dopamine neurons in SNpc were enlarged (magnitude 200 ×). (**B**) The stereological counts of THir DA neurons in the SNpc. *Significantly difference from genotype control, *p* < 0.05.

The pathological hallmark of PD is progressive neuronal loss in the SNpc. We previously demonstrated that a single injection of LPS (5 mg/kg) in WT mice produces PD-like neurodegeneration of dopaminergic neuron loss with motor deficits^2^. The present results showed that LPS (2 mg/kg) and DSP-4 (50 mg/kg) produced THir dopamine neuron loss (Fig. 2A). The stereological counting number of THir dopamine neurons in the SNpc of WT mice was 6,926 ± 1,307, 6,550 ± 1,115 and 4,974 ± 1,062 for Control, PLS, and DSP-4, respectively; The stereological counting number of THir dopamine neurons in the SNpc of SNCA mice was 6,660 ± 1,046, 4,007 ± 1,010 and 3,587 ± 863 for Control, PLS, and DSP-4, respectively (Fig. 2B). DSP-4 significantly decreased THir DA neurons in SNCA mice. Although not statistically significant, low dose of LPS (2 mg/kg) also decreased THir DA neurons in SNCA mice by 40%.

### Colon gene expression

**Figure 3:**
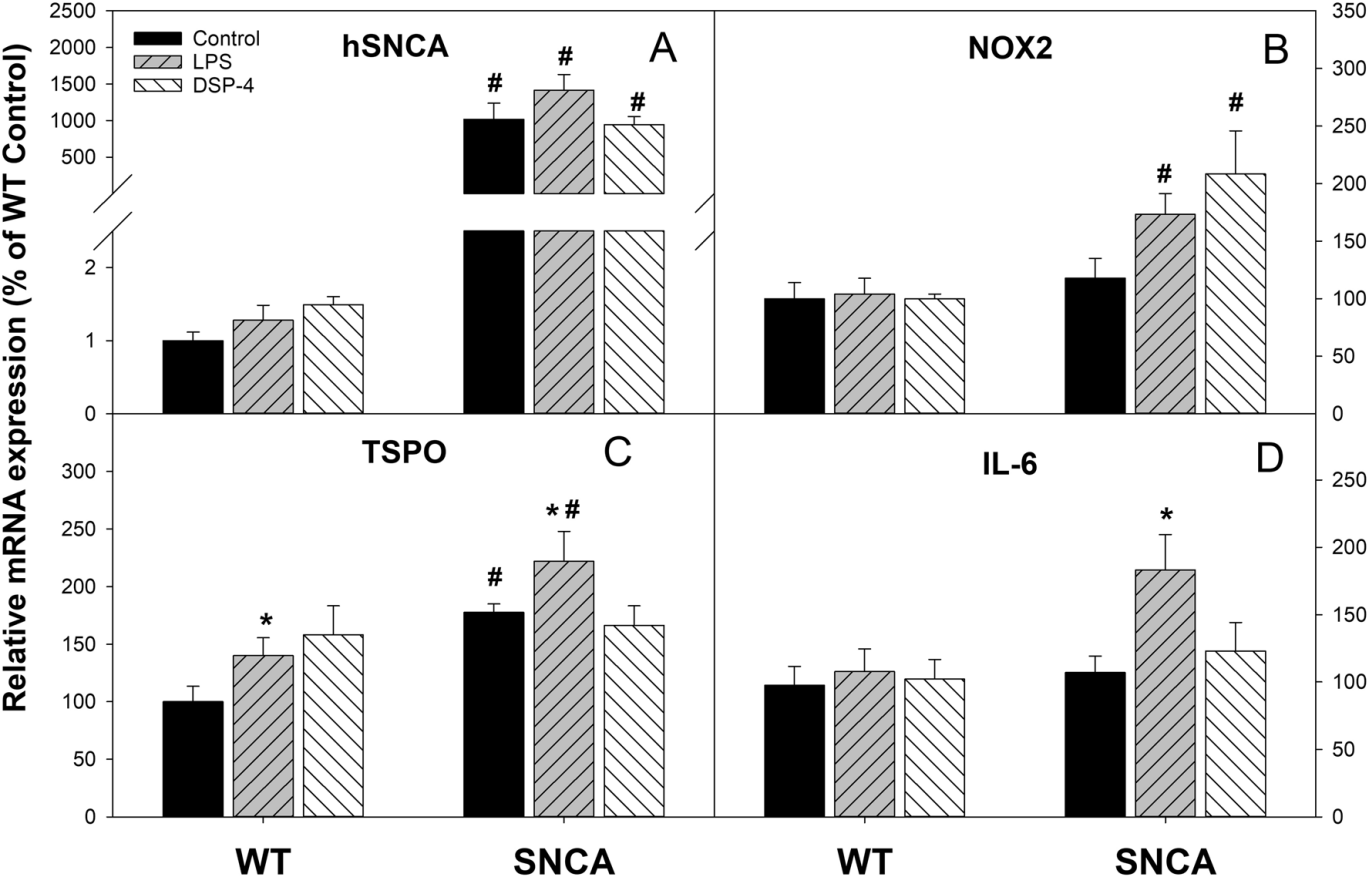
The expression of proinflammatory genes in mouse colon. Mice were given a single injection of LPS (2 mg/kg), or DSP-4 (50 mg/kg), and colon RNA was isolated at the end of experiments (13 months) and subjected to real-time RT-PCR analysis. Data are mean ± SE of 4–7 animals. *Significantly different from Controls, *p* < 0.05; ^#^Significant differences between WT and SNCA mice, *p* < 0.05.

At the end of the experiment, colons were collected to extract RNA for real-time RT-qPCR analysis (Fig. 3). The expression of α-synuclein in the colon was thousand-fold higher in SNCA mice (human mutant and intrinsic α-synuclein gene) than in WT mice (intrinsic α-synuclein only) (1,019 vs 1.0), LPS injection caused a further 40% increase in hSNCA expression in SNCA mice (1,415 vs 1,019) (Fig. 3A). Compared with WT mice, the expression of NOX2, the catalytic subunit of NADPH oxidase which is a critical superoxide-generating enzyme, in SNCA mice was increased 45% (173 vs 118) by LPS and 70% by DSP-4 (208 vs 118), although the results did not reach statistic significant levels compared to SNCA controls, they are significantly different from WT mice treated with LPS or DSP-4 (Fig. 3B); The expression of translocator protein (TSPO, a marker of inflammation) was 1.8-fold higher in SNCA mice and was further increased by LPS. Altogether, SNCA mice had higher expression of TSPO than WT mice (Fig. 3C); The expression of interleukin 6 (IL-6, a proinflammatory cytokine) in SNCA mice was increased 70% by LPS (183 vs 107) (Fig. 3D). However, there were no changes in the gut expression of acute inflammatory mediators TNFα and IL-1β (data not shown).

### Alterations in gut microbiome

**Figure 4:**
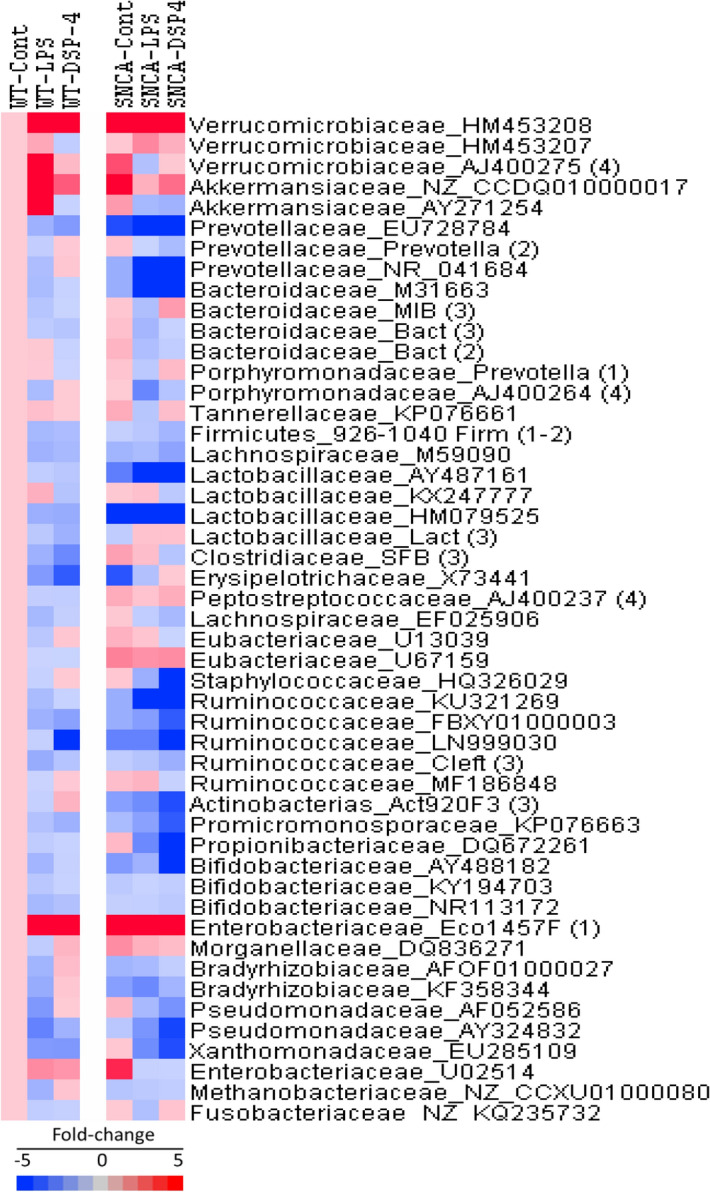
Heatmap of relative abundance of 50 gut microbiota in WT and SNCA mice. Mice were given a single injection of LPS (2 mg/kg), or DSP-4 (50 mg/kg), Colon bacteria DNA was extracted 13 months later and subjected to qPCR analysis with specific 16S rRNA or gene primers for specific microbiome abundance, in a style of Family-Primer access with fold-change. Red indicates increases, and blue indicates decreases.

Colon contents were collected at the end of experiments (13 months) and bacterial DNA was extracted and subjected to PCR amplification with 16S rRNA or relevant gene primers for specific microbiota. We have examined relative abundance of 50 microbiota genus/species from 25 families belonging to 7 phyla as illustrated in Fig. 4 heatmap setting WT control as 1. For easy visual comparisons, the fold-change was used to show increased or decreased abundance in specific microbiota and in the same order as Supplementary Table 2.

### Increases in phylum Verrucomicrobia

**Figure 5:**
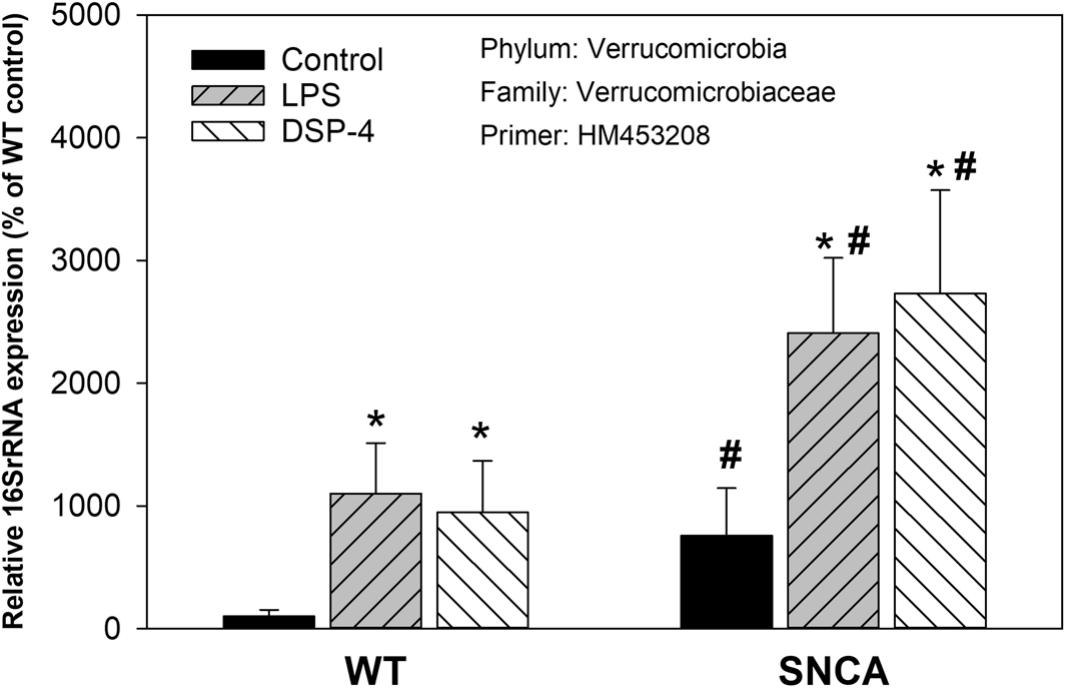
Relative abundance of phylum *Verrucomicrobia*. Mice were given a single injection of LPS (2 mg/kg), or DSP-4 (50 mg/kg), and colon content was collected to extract bacterial DNA and subjected to PCR analysis. Data are mean ± SE of 4–7 animals. *Significantly different from Controls, *p* < 0.05. ^#^Significant differences between WT and SNCA mice, *p* < 0.05;

The relative abundance of *Verrcomicrobia* is shown in Figs. 4 and 5 and Supplemental Table 2. Compared to WT control mice, the relative abundance of *Verrucomicrobiaceae* (Primer: HM453208) was increased 7.6-fold in SNCA control mice; LPS and DSP-4 further increased it to 24- and 27-fold, respectively in SNCA mice, while LPS and DSP-4 only increased it approximately tenfold in WT mice, making it significantly different between SNCA and WT mice (Fig. 5). Among 5 *Verruomicrobia* examined, *Akkermansiaceae* (Primer: NZ_CCDQ010000017) showed higher expression in SNCA mice and in response to LPS or DSP-4. Another *Akkermansiaceae* (Primer: AY271254) and *Verrucomicrobiaceae* (Primer: AJ400275) showed increases in response to LPS; but *Verrucomicrobiaceae* (Primer: HM453207) did not show changes (Fig. 4).

### Decreases in phylum Bacteroidetes

**Figure 6:**
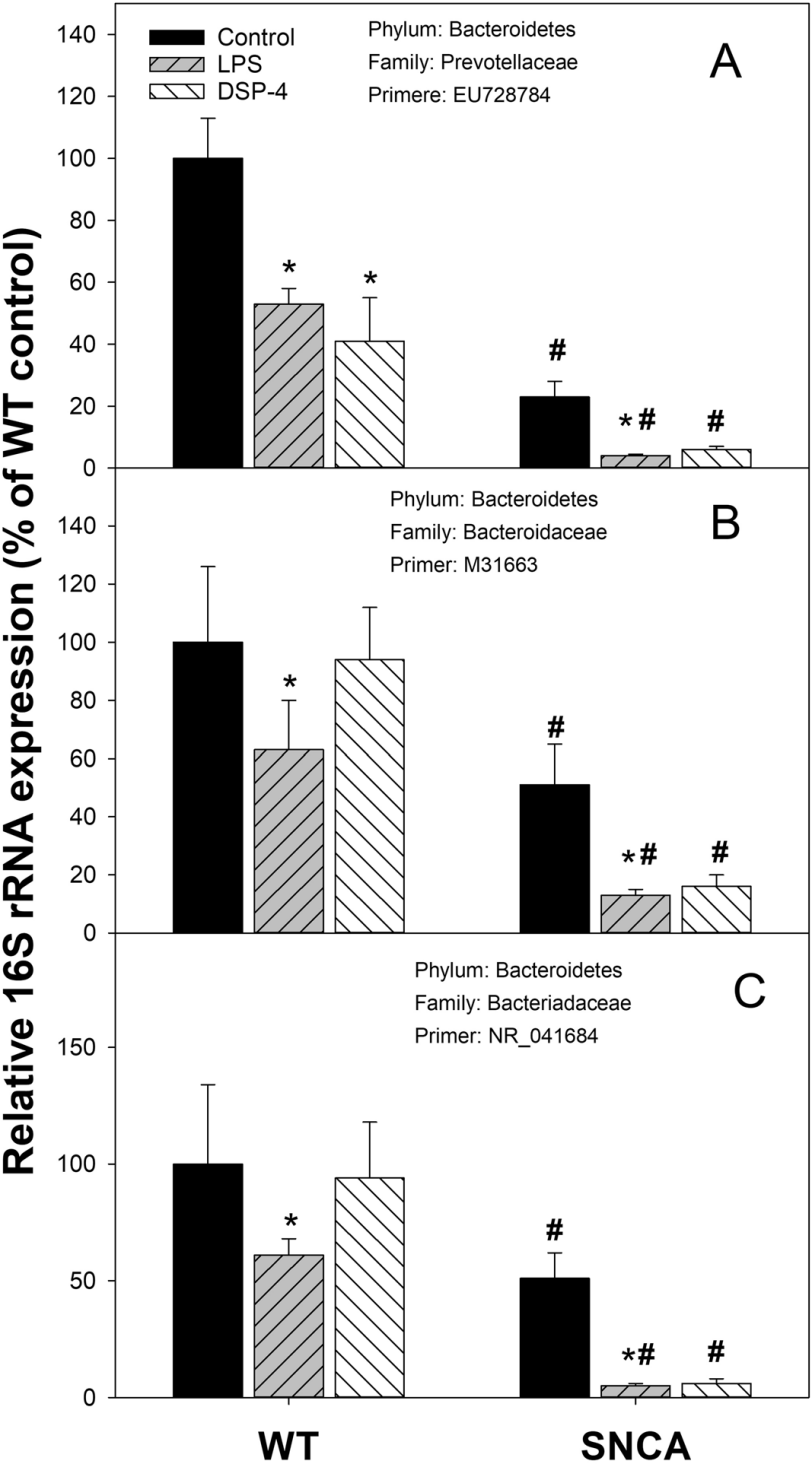
Relative abundance of phylum *Bacteroidetes*. Mice were given a single injection of LPS (2 mg/kg), or DSP-4 (50 mg/kg), and colon content was collected to extract bacterial DNA and subjected to PCR analysis. Data are mean ± SE of 4–7 animals. *Significantly different from Controls, *p* < 0.05; ^#^Significant differences between WT and SNCA mice, *p* < 0.05.

The relative abundance of *Bacteroidetes* is shown in Figs. 4, 6 and Supplemental Table 2. Compared to WT control, the relative abundance of *Prevotellaceae* (Primer: EU728784) was decreased 75% in SNCA control mice, while LPS or DSP-4 produced more decreases in both WT and SNCA mice (Fig. 6A). The relative abundance of *Bacteroidaceae* (Primer: M31663) was also decreased 50% in SNCA control mice in comparison to WT control mice; LPS or DSP-4 produced even greater decreases in SNCA mice (Fig. 6B). Furthermore, the relative abundance of *Prevotellaceae* (Primer: NR_041684) was also decreased 50% in SNCA control mice in comparison to WT control mice, while LPS or DSP-4 produced more decreases in SNCA mice (Fig. 6C). Among 10 *Bacteroidetes* examined, others showed mild decreases (*Prevotellaceae*, Primer: Prevotrlla (2); *Bacteroidaceae*, Primer: Bact (2) and (3); *Porphyromonadaceae*, Primer: AJ400264, Prevotella (1)), while others did not change (*Tannerellaceae*, Primer: KPO76661, *Bacteroidaceae*, Primer MIB (3)) (Fig. 4).

### Mixed changes in phylum Firmicutes

The phylum of *Firmicutes* is the major microbiota in the gut, so we examined the relative abundance of 18 genus/species in this phylum, and the mixed results were obtained (Fig. 4 and Supplemental Table 2). For example, the relative abundance of *Lactobacillaceae* (Primer: HM079525) was decreased 80% in SNCA control mice, while LPS or DSP-4 produced further decreases in SNCA mice, consistent with the observation of depletion of *Lactococcus spp*. in the PD patients^15^ ; However, the relative abundance of *Eubacteriaceae* (Primer: U67159) was increased to 245% in SNCA control mice compared to WT control mice, while LPS or DSP-4 produced similar increases in SNCA mice; Four genera of *Ruminococcaceae* (Primers: KU321269, FBXY01000003, LN999030, Cleft) showed decreases, while one genus of *Ruminococcaceae* (Primer: MF186848) was largely unchanged. Some genera showed mild decreases such as *Lachnospiraceae* Primer: M59090, *Lactobacillacea*e, Primer AY487161, *Erysipelotrichaceae* Primer:X73441 in response to LPS or DSP-4, while others did not change (e.g., *Peptostreptococcaceae*, Primer: AJ400237, *Eubacteriaceae* Primer U13039, U67159) (Fig. 4).

The *Firmicutes/Bacteroidetes* ratio is an important parameter for evaluation of the distribution of gut flora^28,29^. Taken all 18 *Firmicutes* bacteria and 10 *Bacteroidetes* relative abundance into consideration, the *Firmicutes/Bacteroidetes* ratio was increased by LPS in SNCA mice by 165% (Supplementary Table 3).

### Phylum Actinobacteria

**Figure 7:**
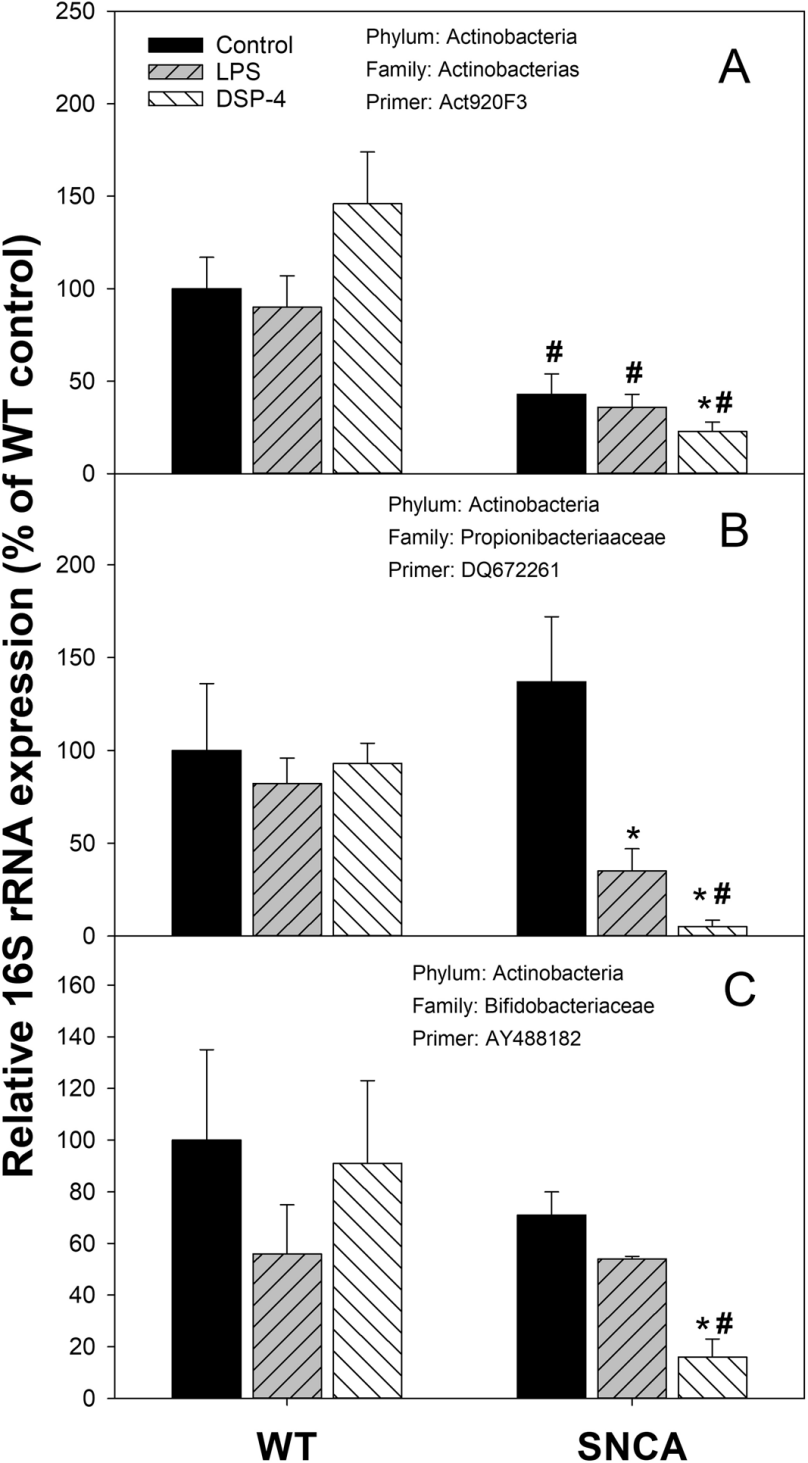
Relative abundance of phylum *Actinobacteria*. Mice were given a single injection of LPS (2 mg/kg), or DSP-4 (50 mg/kg), and colon content was collected to extract bacterial DNA and subjected to PCR analysis. Data are mean ± SE of 4–7 animals. *Significantly different from corresponding genotype Controls, *p* < 0.05; ^#^Significant differences between WT and SNCA mice, *p* < 0.05.

The relative abundance of *Actinobacteria* is shown in Figs. 4, 7 and Supplemental Table 2. The relative abundance of *Actinobacterias* (Primer: Act920)^30^ was decreased 55% in SNCA control mice in comparison to WT control mice, which was further decreased by DSP-4 (Fig. 7A). LPS or DSP-4 also decreased the relative abundance of *Propionibacterium acnes* (Primer: DQ672261)^31^ in SNCA mice only (Fig. 7B). The relative abundance of *Bifidobacteriaceae* (Primer: AY488182) was decreased 30% in SNCA control mice in comparison to WT control mice, while DSP-4 further decreased its expression in SNCA mice (Fig. 7C). Among 6 *Actinobacterias* examined, some showed mild decreases in SNCA mice (e.g., *Promicromonosporaceae* Primers: KPO76663) while others were not significantly changed (e.g., *Bifidobacteriaceae* Primer: NR113172 and KY194703) (Fig. 4).

### Phylum Proteobacteria

**Figure 8:**
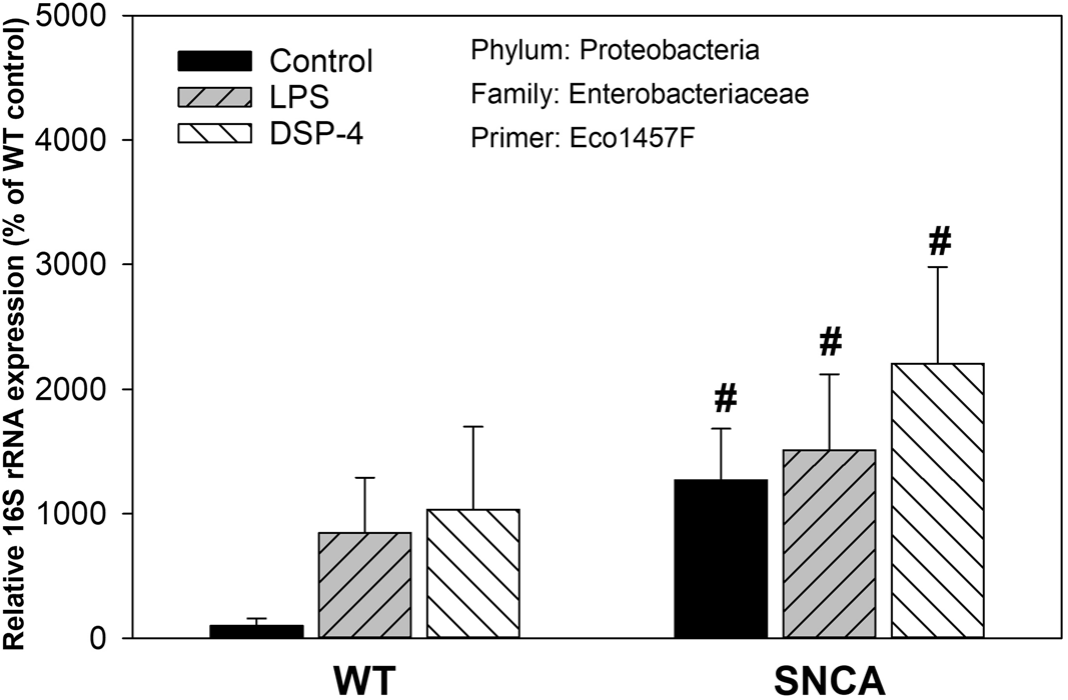
The relative abundance of phylum *Proteobacteria.* Mice were given a single injection of LPS (2 mg/kg), or DSP-4 (50 mg/kg), and colon content was collected to extract bacterial DNA and subjected to PCR analysis. Data are mean ± SE of 4–7 animals. ^#^Significant differences between WT control and SNCA control mice, *p* < 0.05.

The relative abundance of *Proteobacteria* is shown in Fig. 4, 8 and Supplemental Table 2. The expression of *Enterobacteriaceae* (Primer: Eco1457)^30^ was increased 13-fold in in SNCA control mice in comparison to WT control mice, with large individual variations. Both LPS and DSP-4 tended to increase relative abundance of *Enterobacteriaceae* in both WT and SNCA mice but were not reach statistical significance due to large variations. However, SNCA mice had higher levels than WT mice (Fig. 8). Among 8 *Proteobacteria* examined, another *Enterobacteriaceae* (Primer U02514) showed mild increases in SNCA control mice and in response to LPS and DSP-4 in WT mice. Others did not show changes (e.g., *Morganellaceae* Primer DQ836271, *Bradyrhizobiaceae* Primer AF0F1000027, and *Pseudomonadaceae* Primer AY324832), while some others are decreased (*Xanthomonadaceae* Primer EU285109; *Bradyrhizobiaceae* Primer KF358344, and *Pseudomonadaceae* Primer AF052586) (Fig. 4).

### Phylum Euryarchaeota and Fusobacteria

The expressions of Phylum of *Euryarchaeota* (Kingdom: Archaea) and *Fusobacteriaceae* are shown in Fig. 4 and Supplemental Table 2. The *Methanobateriaceae* (*Euryarchaeota*) includes methanogens, which produce methane and are often found in intestines and increased in PD patients (Unger et al., 2016). *Fusobacterium* (*Fusobacteriaceae*) is a genus of anaerobic, Gram-negative, non-sporeforming bacteria, and is often associated with ulcerative colitis. There was no difference between SNCA and WT control mice in relative abundance of *Methanobateriaceae* (Primer NZ_CCXU01000080) and *Fusobacteriu* (Primer NZ_CCXU01000080), nor the differences were found in response to LPS or DSP-4 treatment (Fig. 4).

## Discussion

The present study demonstrated that (1) the “two-hit” mouse PD model (LPS or DSP-4 injected SNCA mice) produced more pronounced effects than “one-hit” model (LPS or DSP-4 injected WT mice). Overexpression of human mutant α-synuclein renders mice vulnerable to LPS or DSP-4 challenge; (2) Both LPS and DSP-4 significantly produced alterations in gut microbiome, with changes in relative abundance in gut microbiome, accompanied by neurodegeneration and motor deficits, suggesting the Gut-Brain-Microbiota disruption is important in PD development.

Compared with our previous mouse model generated by an intraperitoneal injection of a higher dose of LPS (5 mg/kg) into WT mice^2,23^, our present “two-hit” was created by a lower dose of LPS (2 mg/kg) in SNCA or WT mice. SNCA mice had thousand-fold higher expression of α-synuclein than their WT littermates in the gut, which renders SNCA mice much more sensitive to LPS challenge, as evidenced by increased expression of inflammatory mediators (NOX2, TSPO, and IL-6)^24,25^ in the large intestine. The gut as the gateway to the environment with its ENS plays a crucial role in the neurodegenerative process that leads to PD^4^, resulting in motor and non-motor symptoms^32,33^. In this study, the rotarod activity and wirehang were greatly impaired by LPS and DSP-4, especially in SNCA mice. However, despite the comparable mouse body weight was observed in various groups, the overall higher rotarod activity in SNCA groups than WT groups is not clear and needs further investigation. The present study fortified the neurodegeneration by depleting brain NE with DSP-4 (50 mg/kg)^24^, but not with the low dose of LPS (2 mg/kg) in WT mice. However, overexpression of α-synuclein in SNCA mice greatly potentiated LPS (2 mg/kg)- and DSP-4 (50 mg/kg)-induced dopaminergic neuron loss. It’s worth noting that the levels of expression of inflammatory markers in the colon do not reach statistical significance in the present study (Fig. 3). In general, the increase in inflammatory genes expression at chronic inflammation phase is extremely difficult to be detected accurately as they only have mild increase compared to dramatic increase at acute inflammation stage. Mild NOX2 activation plays important roles in LPS and DSP-4 induced low-grade chronic neuroinflammation^24^, and a mild (45–70%) increase in NOX2 in the gut of SNCA mice in the present study could also be important for superoxide production and chronic inflammation in the enteric nervous system similar to the chronic neuroinflammation following LPS/DSP-4 injection^24,26^. Minimally toxic dose of LPS and α-synuclein oligomer elicit synergistic dopaminergic neurodegeneration through microglial NOX2 activation^34^, and the present study demonstrated that overexpression of α-synuclein also predisposes SNCA mice to gut inflammation, motor activity impairment and PD development.

Gut dysbiosis may provoke inflammation, triggering the development of α-synuclein pathology^35^. Gut microbiota dysfunction promoted SNCA-mediated motor deficits and neuroinflammation in another strain of human SNCA mutant mice (ASO)^36^, and when germ-free mice were recolonized with microbiota from PD patients, the Parkinsonism occurred^37^. The use of antibiotics could produce dysbiosis to alter host-bacterial interactions leading to functional changes^29,38,39^. On the other hand, probiotics could alter gut microbiota for beneficial effects leading to the possibility of food-based therapies for PD^40^.

Accumulating evidence indicates the association of gut microbiota with PD in patients from USA^41,42^, Finland^18,20,43^, Germany^16,19,21,44^, Russia^45^, Japan^46^, and China^22,47^. However, the findings are not consistent, and these different results might be dependent on the race, diets, life styles, the location of sample size and collection sites (i.e., nasal, oral, intestinal mucosa, feces); these variations could also be due to the detection methods (PCR vs NGS) and the disease status as commented^48,49^. However, nothing is known about gut microbiota alterations in LPS and DSP-4 in combination of SNCA mutant “two-hit” models. The present study used 50 qPCRs to fill the gap, and the major bacteria composition changes are discussed below.

### Increases in Verrucomicrobia

*Verrucomicrobia* contains only a few described species. *Verrucomicrobia* are Gram-negative, coccoid or rod-shaped bacteria with unusual cellular structure, featuring wart-like cellular protrusions and the unique compartmentalized cell plan. Increased *Verrcumicrobia* are reported in PD patients from Finland^18^ and USA^41^. In PD patients from German, some reports showed increased *Verrcumicrobia*^16,19,21^, while others did not find the association^44^. In the present study, marked increases in *Verrucomicrobia* were observed, and SNCA mice had significantly increased abundance in 3 species (Fig. 4), and LPS and DSP-4 could further increase the abundance of *Verrucomicrobia* in SNCA mice (Fig. 5). A recent meta-analysis study of gut dysbiosis in PD patients indicates that increased *Akkermansiaceae* family of *Verrucomicrobia* is a consistent finding^50^.

### Decreases in Prevotellaceae and Bacteroidetes

*Provotellaceae* is a common bacteria family in the colon related to mucin synthesis in the mucosal layer of the gut and production of health-promoting neuroactive short chain fatty acids through fermentation of soluble fiber^51^, and *Prevotella*-derived hydrogen sulfide^52^ is thought to play a role in PD-related GI symptoms such as constipation and neuroprotection. Decreased *Prevotellaceae* was reported in PD patients^16–19,42,45^. PD patients with IBS had more non-motor symptoms and had a lower *Prevotella*^20^. However, in other reports, *Prevotella* was reported to have no change^42,44,46^ or even increased^41^. In the present study, a marked decrease in abundance of *Prevotella* in SNCA mice and after LPS or DSP-4 treatments was seen with one probe (EU728784), but showed no apparent changes with the general probe for *Prevotella*^30^, or a mild decrease with another reported probe^53^. Thus, the selection of probes could affect the outcome. Nonetheless, the decreases in the abundance of *Prevotellaceae* could be associated with LPS-induced dysfunction in the ENS, especially in SNCA mice.

*Bacteroidetes* are the important phylum of bacteria in the gut. The decreased *Bacteroidetes* was also seen in PD patients from German^19,41^, Russia^45^, and Japan^46^. Among the 10 *Bacteroidetes* species examined, LPS decreased the abundance of 6 species, while DSP-4 decreased the abundance of 4 species, especially in SNCA mice (Fig. 4 and Supplementary Table 3), indicating the decreases in *Bacteroidetes* are associated with the “two-hit” mouse PD models.

### Increased Firmicutes/Bacteroidetes ratios

The *Firmicutes* are the most abundant bacteria in the gut, most of which have Gram-positive cell wall structure. They have round cells, called cocci, or rod-like forms (bacillus). In PD patients, the abundance of *Firmicutes* was reported to have no change^19^, increased^41^, or decreased^19,41,50^, depending on the bacteria species examined^49^. The increased *Firmicutes/Bacteroidetes* ratio was also observed in many neurodegenerative diseases, including Alzheimer’s disease^28,29^. In the present study, we have examined 18 species from 7 families of the *Firmicutes* phylum. Some were increased, while others were decreased, and most of them showed no significant changes. It should be noted that LPS and DSP-4 produced more alterations in SNCA mice than in WT mice. When taken the *Firmicutes/Bacteroidetes* ratio into consideration, LPS treatment markedly increased the ratio (165%) in SNCA mice, indicating the susceptibility of the “two-hit” model.

### Decreases in Actinobacteria

*Actinobacteria* was reported to be lower in PD patients^17^, and in rotenone-induced PD animal models^54^, and are susceptible to age-related alterations^55^. In the present study, LPS and DSP-4 decreased the expression of *Actinobacteria* in SNCA mice (Fig. 7), indicating the susceptibility of the “two-hit” model.

### Increases in Enterobacteriaceae

*Enterobacteriaceae* belongs to *Proteobacteria,* a major phylum of Gram-negative bacteria. They include a wide variety of pathogens, such as *Escherichia, Salmonella, Vibrio, Helicobacter, Yersinia*, and many other notable genera. *Enterobacteriaceae* were reported to increase in PD patients^19,41,44^, and in PD mouse models^56^. Oral administration of *Proteus mirabilis* (Enterobacteriaceae) isolated from PD mice significantly induced motor deficits, dopaminergic neuronal damage and neuroinflammation, and stimulated α-synuclein aggregation in the brain as well as in the colon, probably in association of LPS-induced inflammation in the gut^56^. In the current study, marked increases in *Enterobacteriaceae* were seen in SNCA mice, and in LPS and DSP-4 treated WT mice (Fig. 8). A huge individual variation was seen in its expression. *Enterobacteriaceae* is thought to be putative pathobionts^47,56^. How the increased *Enterobacteriaceae* might contribute the LPS/DSP-4 induced inflammation and SNCA pathology in the gut warrants further investigation.

Compared to 16S DNA sequencing, qPCR has also been used for bacteria 16S rRNA quantification^57^, such as the studies with PD toxicant rotenone^58^, intestinal inflammation^59^, dietary Xylitol^53^, fecal short chain fatty acids in relation to microbiota in PD patients^19^, and antibiotics in relation to bile acid metabolism and microbiota^29^. The current study using these specific primers could shed light on gut microbiota alterations in “two-hit” PD models. Indeed, overexpression of α-synuclein in SNCA mice aggravated LPS- and DSP-4-induced inflammation and α-synucleinopathy in the large intestine, accompanied by impaired motor activity and ascending neurodegeneration in the brain in a time-dependent, progressive manner (Song et al., manuscript in preparation).

In summary, this study demonstrated that a ‘two-hit” PD mouse model (SNCA mice given LPS or DSP-4 injection) were more susceptible to PD development than “one-hit” PD model (WT mice given LPS or DSP-4 injection), as evidenced by motor dysfunction, dopaminergic neuron loss, inflammatory gene expression in the colon, and the disruption of gut microbiota. The decreased abundance of *Prevotellaceae* and increased abundance of *Verrucomicobiaceae* are largely in the line with gut microbiota alterations reported in PD patients, and coincides with findings in recent reviews of over 100 publications in PD patients and PD models^49,50^. This study provides evidence that α-synuclein and toxicants interactions are important for PD development.

## Materials and methods

### Chemicals and reagents

Bacterial endotoxin LPS (Sigma-Aldrich, USA), the NE-depleting neurotoxin DSP-4 (Sigma-Aldrich, USA) were reagent grade. All the primers were synthesized by Integrated DNA Technology (Coralville, IA); RNA Mini kit and QIAamp DNA Stool Mini kit (51,504) were from Qiagen (Germantown, MD); Antibody against tyrosine hydroxylase (TH, rabbit: AB152) was from EMD Millipore (Temecula, CA).

### Animals and exposures

A53T α-Syn (B6C3F1/J-Tg-Prnp/SNCA*A53T/83Vle/J) heterozygous breeders (Jackson Laboratories, Bar Harbor, ME, USA) were used to produce A53T homozygous (SNCA) and Non-Tg littermates (WT). All mice were genotyped by SeqWright-DNA-Technology (Houston,TX, USA), and defined as WT (< 0), heterozygous (1–20) and homozygous (> 20) of α-synuclein mutant. Animals were mix-housed in their home cages in the same shelf of the AAALAC accredited animal facilities at the National Institute of Environmental Health Sciences/NIH, with standard temperature (70 ± 2 °F), 12 h light:dark cycle (8:00 – 8:00) and fed on standard rodent chow and tap water ad libitum. However, only homozygous mutant (SNCA) mice and their littermate WT mice were used in the present study. Eight-week-old male WT and SNCA mice received an intraperitoneal injection of LPS (*Escherichia coli* 0111:B4; 2 mg/kg) (WT control n = 4, WT LPS, n = 6, WT DSP-4, n = 7; SNCA control n = 5, SNCA LPS n = 7, SNCA DSP-4 n = 7). We have previously identified that female mice are less responsive to LPS injection and repeated injections of LPS were required to produce dopaminergic neuron loss in female mice (Liu et al., 2008). Therefore, only male mice were used in the present study. The reason for using smaller dose of LPS (2 mg/kg) compared with our previous studies (5 mg/kg) was because SNCA mice are more susceptible to LPS^27^ and are used as a PD model^60^. Mice were euthanized 13 months later. Our previous studies indicate that at this time point, toxin-injected mice display dopaminergic neuron loss in the substantial nigra and motor/non-motor deficits^2,24,25^. Animal study protocols were approved by the Institutional Animal Care and Use Committee of NIEHS, and all methods were performed in accordance with the relevant guidelines and regulations.

### Behavioral tests

The motor coordination activity was performed using rotarod test with a Rotamex device (Columbus Instruments, USA). The parameters of the rotarod system include start speed, acceleration and highest speed (1 rpm, accelerate 12 rpm/2 s, 50 rpm). The mice underwent three consecutive trials. The rest period between each trial was 30 min. The mean latency time to fall off the rotating rod in the three trials was used for the analysis. Grip strength was evaluated using the wirehang test. Mice were suspended by their paws from a wire 20 cm above a soft bedding cushion. Mean latency to fall over the three trials was analyzed. We have performed behavioral tests bi-monthly, including the open-field tests^26^, which will be detailed in another publication (Song et al., manuscript in preparation). In the present study, only the motor function tests at the 13-month time point that matched the time point for gut microbiota analysis was presented.

### Immunohistochemistry

Immunostaining was performed as described previously^24^. Mouse brains were cut into 35 µm sections on a horizontal sliding microtome. The free-floating sections were immune-blocked with 4% goat serum in 0.25% triton/PBS for 1 h. Dopaminergic neurons were stained with anti-tyrosine hydroxylase (TH, 1:4,000, overnight at 4 °C). Sections were incubated with biotinylated secondary antibody for 1 h followed by incubation with Vectastain ABC reagents (Vector Labs, Burlingame, CA) for 40 min and then color was developed with 3,3-diaminobenzidine. To monitor DA neuro degeneration, two individuals blind to the treatment counted the number of TH-immunoreactive (TH-IR) neurons in the SN pars compacta (SNpc) of six evenly spaced brain sections from a series of 24 sections that covered the entire SN^26^. Stereological counts of THir SN*pc* neurons were estimated using an optical fractionator method on an Olympus BX50 stereological microscope within user-defined boundaries^24^. All immunohistochemistry images were captured by a Leica Aperio AT2 Scanner.

### Total RNA and real time RT-PCR analysis

Total RNA was isolated with Qiagen RNA mini kit. The quality and quantity of RNA were determined by NanoDrop (ThermoFisher Scientific, Waltham, MA, USA), with 260/280 > 1.8. Total RNA was reverse transcribed with MuLV reverse transcriptase and oligo-dT primers and real-time PCR analysis using SYBR green PCR master mix, and primers were designed with Primer3. The Ct values were used to calculate the relative expression by the 2^−△△Ct^ method and normalized with β-actin, setting control as 100%.

### Bacteria DNA isolation and PCR analysis

The entire colon contents were collected and total bacteria DNA was extracted with QIAamp Stool DNA extraction kit, with 260/280 > 1.9. The 16S rRNA or relevant gene primers were used to determine the abundance of specific bacteria or archaea. The Total^30^ bacteria were used to normalize the relative abundance of gut microbiota. Primers used to examine gut microbiome were from published primers^19,30,53,59^ or designed with “Primer3” from the internet based on interested microbiota from the literature^18,41,42^, and were listed in Supplementary Table 1, and the raw results were included in Supplementary Table 2. The TreeView version 1.6 (https://treeview.software.informer.com/1.6/) was used to generate a heatmap for visualization.

### Statistics

All data were expressed as mean ± SEM. Comparison of multiple groups was performed using one-way ANOVA analysis followed by GraphPad Prism 8 (GraphPad Software, La Jolla, CA), and *p* < 0.05 was considered significantly different.

## Supplementary information

Supplementary information.

## Data Availability

The datasets generated during and/or analyzed during the current study are available from the corresponding author on reasonable request.
