# Supplementary material for: Norepinephrine depleting toxin DSP-4 and LPS alter gut microbiota and induce neurotoxicity in α-synuclein mutant mice
Source: Sci Rep. 2020 Sep 14;10:15054. doi: 10.1038/s41598-020-72202-4 (PMC7490385; doi:10.1038/s41598-020-72202-4)
Supplement: Supplementary file 1 — Supplementary information. [file 41598_2020_72202_MOESM1_ESM.pdf]

Sci Rep- ID 82e1836f-54ac-4947-b2a2-45d5c497c9dc- Supplementary Table 1-3

Norepinephrine depleting toxin DSP-4 and LPS alter gut microbiota and induce neurotoxicity in α-synuclein mutant mice

Sheng Song[1], Jie Liu[1,2], Feng Zhang[2], Jau-Shyong Hong[1]

[1]Neuropharmacology Section, Neurobiology Laboratory, National Institute of Environmental Health Sciences, National Institutes of Health, Research Triangle Park, North Carolina, 27709, USA.

[2]Key Lab for Basic Pharmacology of Ministry of Education and Joint International Research Laboratory of Ethnomedicine, Zunyi Medical University, Zunyi, Guizhou, 563000, China

Running Title: Gut microbiota in LPS/DSP-4 treated SNCA mice

Send Correspondence to

Jie Liu, Jie@liuonline.com

JS Hong, Hong3@niehs.nih.gov

Supplemental Table 1. primer sequences for qPCR

Gene access #	Forward	Reverse
HM453208	GTCATTCCTCGGTGTGGATT	TTCATGTCCATGTCGTCCAT
HM453207	TGACCATGTCGTTGTCCTGT	TTTGCATGGACCCTTTCTTC
AJ400275 (4)	ATCATTCACAATGGGGGAAA	GGCACAGAGTTAGCCGTCTC
NZ_CCDQ010000017	GCTGTTCCATAGGGGGAGTA	AAACCAGTGGCAACAAGGTC
AY271254	ATCATTCACAATGGGGGAAA	GGCACAGAGTTAGCCGTCTC
EU728784	CGAAAGGTTTAGCGGTGAAG	CGTAGGAGTTTGGACCGTGT
Prevotella (2)	CATGACGTTACCCGCAGAAGAAG	TCCTGCACGCTACTTGGCTG
M31663	GACACGTGTACCCAATGCAG	GGCAACCTGAGCCTCTGTAG
NR_041684	GCAGCAGTTGAACAACCAGA	ATGCGTATGCCTTTTTGGTC
MIB (3)	CCAGCAGCCGCGGTAATA	CGCATTCCGCATACTTCTC
Bact (3)	GGTTCTGAGAGGAGGTCCC	GCTGCCTCCCGTAGGAGT
Bact (2)	GGARCATGTGGTTTAATTCGATGAT	AGCTGACGACAACCATGCAG
Prevotella (1)	GGTGTCGGCTTAAGTGCCAT	CGGACGTAAGGGCCGTGC
AJ400264 (4)	GAACCCGAAAACCTGTCTCA	TTTACCCTAGGGCGTTCCTT
KP076661	ACACGGACCAGACTCCTACG	ACACGTCCCGCACTTTATTC
926-1040 Firm (1-2)	GGAGYATGTGGTTTAATTCGAAGCA	AGCTGACGACAACCATGCAC
M59090	AGTAACGCGTGGGTAACCTG	ACCGGAGTTTTTCACACCAG
AY487161	GGGTCAATTGCAGGAAAGAA	GTTGATGGAGCTGGGTCAGT
KX247777	TTTGAGTGAGTGGCGAACTG	CCAAAAGTGATAGCCGAAGC
HM079525	CCCAAGAGATCGGGATAACA	AGGCCGTTACCCTACCAACT
Lact (3)	AGCAGTAGGGAATCTTCCA	CACCGCTACACATGGAG
SFB (3)	GACGCTGAGGCATGAGAGCAT	GACGGCACGGATTGTTATTCA
X73441	AGGATCGTGGAATTCCTGTG	CGGCGTGGACTACTAGGGTA
AJ400237 (4)	GGTAACGGCCTACCAAGTCA	GTGCAATATTCCCCACTGCT
EF025906	CGCAGCAAACGCAATAAGTA	TAAGGTTCTTCGCGTTGCTT
U13039	TAACGGCTTACCAAGGCAAC	GTGCAATATTCCCCACTGCT
U67159	CCCTTATGACCTGGGCTACA	CAATCCGAACTGGGATCTGT
HQ326029	ACCAGTACCACCTGCCAAAG	TGGTGCCACTGCTTTAACCT
KU321269	ATATTGCACAATGGGGGAAA	AGCCGGAGCTTTCTCCTTAG
FBXY01000003	TGCTCCAAGCAGCTCTACAA	CTGCCGTATTCGGAAAACAT
LN999030	GGAACAGCCGTACAGGGTTA	GAACACGAAGAACGCCAAAT
Cleft (3)	GTTGACAAAACGGAGGAAGG	GACGGGCGGTGTGTACAA
MF186848	GTGAGGAACCTGCCTCAAAG	GGCCGTTACCTCACCAACTA
Act920F3 (3)	TACGGCCGCAGGCTA	TCRTCCCCACCTTCCTCCG
KP076663	AGCTTGTTGGTGGGGTAATG	GTGCAATATTCCCCACTGCT
DQ672261	TGGGGTAGTGGCTTACCAAG	GTGCAATATTCCCCACTGCT
AY488182	AGGGTCTGAAGAACGTGGTG	TTGCAGCAATCTGTTCCTTG
KY194703	TCGGGGTGAGTGTACCTTTC	GCCCTTTACGCCCAATAAAT
NR113172	GCCCGTCAAGTCATGAAAGT	CCGGTACGGCTACCTTGTTA
Eco1457F (1)	CATTGACGTTACCCGCAGAAGAAGC	CTCTACGAGACTCAAGCTTGC
DQ836271	GCATCACTGATCCCATTCCT	TGAGTCAACCGCTACAGCAC
AFOF01000027	CGAGTCGATGTGCAGTGTG	TCAGCTACACCAACGGTGAC
KF358344	AGCCACATTGGGACTGAGAC	CAACCCTAAGGCCTTCATCA
AF052586	AGAACTCCAGGTGCCCTCTT	AGAAGCAGCGTTCGGTGTAT
AY324832	GCGGTAAAGCCTGAAAACAG	CTCATGACGAGGGTTCGATT
EU285109	GAGGAATTCACCCAGGTGCT	GTGATCGGGTGGCTCTTGT
U02514	GATTGTCGCTCTTCCAGCTC	GTCCGCAACCGTTGAGTAAT
NZ_CCXU01000080	CGAGTTAACCAGTCCCAAGC	CATTAGGACAGGGTGCAGGT
NZ_KQ235732	GGAGCCACTATCGACTACGC	GTCCCTGATGGTCGTCATCT
Eub338-518 (1-3)	ACTCCTACGGGAGGAGCAG	ATTACCGCGGCTGCTGG

(1) Medina et al., 2017; (2) Uebanso et al., 2017; (3) Wellman et al., 2017; (4) Zhang et al., 2014

Supplementary Table 2. Relative expression of 16S rRNA in the "Two-hit" mouse models

			WT littermate			SNCA		
**Phylum**	**Family**	**Gene access #**	**Cont**	**LPS**	**DSP4**	**Cont**	**LPS**	**DSP4**
Verrucomicrobia	Verrucomicrobiaceae	HM453208	100 ± 53	1100 ± 412	947 ± 422	760 ± 386	2410 ± 615	2930 ± 1040
	Verrucomicrobiaceae	HM453207	100 ± 15	170 ± 63	83 ± 27	107 ± 14	236 ± 58	154 ± 38
	Verrucomicrobiaceae	AJ400275 (4)	100 ± 34	977 ± 466	134 ± 86	348 ± 162	67 ± 53	106 ± 40
	Akkermansiaceae	NZ_CCDQ010000017	100 ± 47	1714 ± 735	321 ± 200	865 ± 331	153 ± 111	283 ± 115
	Akkermansiaceae	AY271254	100 ± 55	937 ± 492	93 ± 79	195 ± 104	58 ± 52	54 ± 18
Bacteroidetes	Prevotellaceae	EU728784	100 ± 13	53 ± 5	41 ± 14	23 ± 5	4 ± 0.5	6 ± 1
	Prevotellaceae	Prevotella (2)	100 ± 18	86 ± 4	103 ± 24	115 ± 15	97 ± 7	61 ± 9
	Prevotellaceae	NR_041684	100 ± 34	61 ± 7	94 ± 24	51 ± 11	5 ± 1	6 ± 2
	Bacteroidaceae	M31663	100 ± 26	63 ± 17	110 ± 42	51 ± 14	13 ± 2	16 ± 4
	Bacteroidaceae	MIB (3)	100 ± 20	72 ± 11	97 ± 32	110 ± 33	65 ± 15	193 ± 52
	Bacteroidaceae	Bact (3)	100 ± 28	80 ± 11	72 ± 18	122 ± 37	57 ± 9	94 ± 16
	Bacteroidaceae	Bact (2)	100 ± 12	106 ± 6	93 ± 19	144 ± 27	64 ± 8	83 ± 10
	Porphyromonadaceae	Prevotella (1)	100 ± 9	103 ± 9	98 ± 14	112 ± 15	77 ± 6	133 ± 10
	Porphyromonadaceae	AJ400264 (4)	100 ± 25	61 ± 12	102 ± 39	101 ± 20	34 ± 9	70 ± 24
	Tannerellaceae	KP076661	100 ± 16	125 ± 30	101 ± 12	160 ± 15	73 ± 15	127 ± 26
Firmicutues	Firmicutes	926-1040 Firm (1-2)	100 ± 28	58 ± 5	62 ± 12	88 ± 15	74 ± 8	56 ± 7
	Lachnospiraceae	M59090	100 ± 11	54 ± 6	59 ± 17	55 ± 4	61 ± 20	45 ± 15
	Lactobacillaceae	AY487161	100 ± 45	84 ± 38	74 ± 9	32 ± 8	17 ± 7	11 ± 2
	Lactobacillaceae	KX247777	100 ± 26	154 ± 61	71 ± 9	105 ± 16	121 ± 60	77 ± 11
	Lactobacillaceae	HM079525	100 ± 18	52 ± 16	50 ± 19	20 ± 7	3 ± 1	8 ± 2
	Lactobacillaceae	Lact (3)	100 ± 17	76 ± 22	53 ± 16	82 ± 17	117 ± 31	119 ± 42
	Clostridiaceae	SFB (3)	100 ± 35	53 ± 28	35 ± 21	184 ± 88	126 ± 16	72 ± 43
	Erysipelotrichaceae	X73441	100 ± 39	41 ± 19	25 ± 10	24 ± 19	67 ± 19	104 ± 25
	Peptostreptococcaceae	AJ400237 (4)	100 ± 20	86 ± 13	83 ± 16	162 ± 39	115 ± 19	158 ± 31
	Lachnospiraceae	EF025906	100 ± 28	58 ± 7	88 ± 29	105 ± 18	83 ± 9	59 ± 8
	Eubacteriaceae	U13039	100 ± 23	75 ± 15	110 ± 25	151 ± 21	111 ± 16	95 ± 12
	Eubacteriaceae	U67159	100 ± 21	96 ± 17	95 ± 27	245 ± 35	213 ± 29	221 ± 19
	Staphylococcaceae	HQ326029	100 ± 18	91 ± 11	104 ± 15	107 ± 22	52 ± 9	15 ± 2
	Ruminococcaceae	KU321269	100 ± 34	61 ± 7	94 ± 24	51 ± 11	5 ± 1	6 ± 1
	Ruminococcaceae	FBXY01000003	100 ± 48	53 ± 13	44 ± 9	51 ± 8	42 ± 9	25 ± 6
	Ruminococcaceae	LN999030	100 ± 58	89 ± 34	16 ± 15	33 ± 21	33 ± 21	6 ± 6
	Ruminococcaceae	Cleft (3)	100 ± 30	50 ± 10	70 ± 13	82 ± 9	71 ± 11	52 ± 4
	Ruminococcaceae	MF186848	100 ± 27	96 ± 34	103 ± 34	126 ± 43	147 ± 31	91 ± 16
Actinobacteria	Actinobacterias	Act920F3 (3)	100 ± 17	90 ± 17	146 ± 28	43 ± 11	36 ± 7	23 ± 5
	Promicromonosporaceae	KP076663	100 ± 26	68 ± 20	53 ± 16	61 ± 13	44 ± 5	25 ± 6
	Propionibacteriaceae	DQ672261	100 ± 36	82 ± 14	93 ± 11	137 ± 35	35 ± 12	5.1 ± 3.4
	Bifidobacteriaceae	AY488182	100 ± 35	56 ± 19	91 ± 32	41 ± 9	54 ± 1	16 ± 7
	Bifidobacteriaceae	KY194703	100 ± 35	74 ± 24	93 ± 26	77 ± 30	94 ± 19	83 ± 29
	Bifidobacteriaceae	NR113172	100 ± 37	58 ± 13	67 ± 20	88 ± 14	87 ± 17	77 ± 5
Proteobacteria	Enterobacteriaceae	Eco1457F (1)	100 ± 60	848 ± 277	1034 ± 666	1270 ± 416	1513 ± 609	2208 ± 773
	Morganellaceae	DQ836271	100 ± 25	82 ± 16	143 ± 37	221 ± 38	153 ± 64	131 ± 60
	Bradyrhizobiaceae	AFOF01000027	100 ± 34	55 ± 10	130 ± 45	55 ± 12	60 ± 19	85 ± 31
	Bradyrhizobiaceae	KF358344	100 ± 30	50 ± 17	110 ± 25	43 ± 5	35 ± 4	56 ± 15
	Pseudomonadaceae	AF052586	100 ± 28	40 ± 13	100 ± 28	143 ± 55	59 ± 17	38 ± 12
	Pseudomonadaceae	AY324832	100 ± 29	33 ± 7	54 ± 14	74 ± 13	39 ± 4	22 ± 6
	Xanthomonadaceae	EU285109	100 ± 25	41 ± 7	40 ± 7	108 ± 32	36 ± 5	23 ± 4
	Enterobacteriaceae	U02514	100 ± 28	228 ± 99	206 ± 97	427 ± 122	91 ± 27	94 ± 27
Euryarchaeota	Methanobacteriaceae	NZ_CCXU01000080	100 ± 27	54 ± 5	111 ± 33	72 ± 13	78 ± 6	84 ± 14
Fusobacteria	Fusobacteriaceae	NZ_KQ235732	100 ± 34	84 ± 11	90 ± 15	104 ± 27	66 ± 6	115 ± 20

Data are mean ± SE of 4-7 animals. (1) Medina et al., 2017; (2) Uebanso et al., 2017; (3) Wellman et al., 2017; (4) Zhang et al., 2014

Supplementary Table 3: Firmicutes/Bacteroids ratio								
				WT littermate			SNCA	
**Phylum**	**Family**	**Gene access**	**Cont**	**LPS**	**DSP4**	**Cont**	**LPS**	**DSP4**
Bacteroidetes	Prevotellaceae	EU728784	100	53	41	23	4	6
	Prevotellaceae	Prevotella	100	86	103	115	97	61
	Prevotellaceae	NR_041684	100	61	94	51	5	6
	Bacteroidaceae	M31663	100	63	110	51	13	16
	Bacteroidaceae	MIB (1)	100	72	97	110	65	193
	Bacteroidaceae	Bact (1)	100	80	72	122	57	94
	Bacteroidaceae	Bact (2)	100	106	93	144	64	83
	Porphyromonadaceae	Prevotella	100	103	98	112	77	133
	Porphyromonadaceae	AJ400264	100	61	102	101	34	70
	Tannerellaceae	KP076661	100	125	101	160	73	127
Firmicutues	Firmicutes	926-1040 Firm	100	58	62	88	74	56
	Lachnospiraceae	M59090	100	54	59	55	61	45
	Lactobacillaceae	AY487161	100	84	74	32	17	11
	Lactobacillaceae	KX247777	100	154	71	105	121	77
	Lactobacillaceae	HM079525	100	52	50	20	3	8
	Lactobacillaceae	FJ462287	100	76	53	82	117	119
	Clostridiaceae	SFB	100	53	35	184	126	72
	Erysipelotrichaceae	X73441	100	41	25	24	67	104
	Peptostreptococcaceae	AJ400237	100	86	83	162	115	158
	Lachnospiraceae	EF025906	100	58	88	105	83	59
	Eubacteriaceae	U13039	100	75	110	151	111	95
	Eubacteriaceae	U67159	100	96	95	245	213	221
	Staphylococcaceae	HQ326029	100	91	104	107	52	15
	Ruminococcaceae	KU321269	100	61	94	51	5	6
	Ruminococcaceae	FBXY01000003	100	53	44	51	42	25
	Ruminococcaceae	LN999030	100	89	16	33	33	6
	Ruminococcaceae	Cleft (2)	100	50	70	82	71	52
	Ruminococcaceae	MF186848	100	96	103	126	147	91
			Firm	73.7222	68.667	94.611	81	67.778
			Bact	81	91.1	98.9	48.9	78.9
			Ratio	0.91015	0.7538	0.9566	1.6564	0.859
